# John B King, FRCS: 8 June 1944 - 6 October 2018

**DOI:** 10.1186/s13018-018-1027-3

**Published:** 2018-12-11

**Authors:** Nicola Maffulli

**Affiliations:** 1 University of Salerno Medical School, Salerno, Italy; 2 Centre for Sport and Exercise Medicine, Queen Mary University of London, London, UK

We got together for Prof. John B King’s funeral on 26 October 2018 in Thurlestone, near Kingsbridge, Devon. Prof. King had a full life and died of complications following aortic aneurism surgery.

Prof. King’s name has been inseparable from that of the Queen Mary University of London for many years. John King qualified at the then London Hospital Medical College in 1967. He completed his orthopaedic training on the London Hospital programme, with time spent in the knee unit at the Royal National Orthopaedic Hospital. Prof. King was a precursor: at a time when British orthopaedic surgery was open only to ideas from the other side of the pond, he understood that the other side of the channel had much to offer, and he spent time in Lyon on a fellowship with Professor Albert Trillat, one of the foremost knee surgeons in the globe.

Prof. King was appointed Senior Lecturer in Orthopaedic and Trauma Surgery to the London Hospital Medical College (precursor to Barts and the London School of Medicine and Dentistry, Queen Mary University) and Honorary Consultant in Orthopaedic and Trauma Surgery to the London Hospital and held this position from 1977 to 2001.

Prof. King’s activity as a trainer in trauma and orthopaedic surgery has brought him in contact with a host of unruly young registrars to whom he communicated the calm feeling of the man in charge and the knowledge that he was always there for them. He has produced a cohort of consultant trauma and orthopaedic surgeons of the highest calibre, who retain the utmost respect for the man who introduced the art and the science of arthroscopy in this part of the world.

Prof. King was a man of great vision and a hard taskmaster. When I first reached the UK from Italy, going through a booklet produced by the General Medical Council, I discovered that the only place in the UK where one could learn sports medicine was the London Hospital. In 1981, the then Mr. John King started, with the help of the powers that be of the College, the Diploma Course in Sports Medicine. It soon became ‘the course’; subsequently, the Academic Department of Sports Medicine was founded, and it is now the Centre of Sport and Exercise Medicine at Barts and the London School of Medicine and Dentistry, Queen Mary University of London. Even in the early part of this century, the course remained the only way in the whole of the British Commonwealth to learn in a concerted fashion sports medicine. The diploma course became the Master of Science in Sport and Exercise Medicine, and it has a younger brother in the Intercalated Bachelor of Science in Sport and Exercise Medicine.

I met John King in person for the first time at the interview for the position of Lecturer in Sports Medicine and Registrar in Orthopaedics at the Institute of Child Health and the Hospital for Sick Children in London, with some sessions at the London Hospital. I remember that day well: with Mr. King, Prof. Phillip Graham, Prof. Michael Preece, Prof. June Lloyd and Mr. Stephen Rowley sat on the formidable panel. I was at the time a basic surgical trainee, wishing to pursue a career in orthopaedics, strongly biased towards sports medicine. My consultant at the time, Mr. JP Green, in Boston, Lincs, told me that I stood little chance to get the job. I was young, inexperienced and not trained in the UK, and sports medicine was but a little part of trauma and orthopaedics. His reference must have said good things about me, and I was offered the position. I remember that Mr. King came out of the interview room and, given my past as a researcher in molecular biology, with a great smile congratulated me and told me ‘Next time we see, you will tell me about Western blotting’. I made sure that I knew about it, but Mr. King never asked me again.

Following that interview, Mr. King supervised my PhD at the Institute and started training me in arthroscopy and sports medicine. I remember that we performed the first arthroscopy of the ankle together, in 1987, and, a few weeks later, we had a look inside the shoulder. The knee was commonplace, and there were times when he undertook 30 operative knee arthroscopies per week.

I was examined by Mr. King more than I like to remember: he was my internal examiner for my Master of Surgery thesis, my external examiner for my MD thesis and the examiner for my Diploma in Sports Medicine, both in Edinburgh and at the Apothecaries. He appointed me as a registrar in the North East Thames Orthopaedic Rotation, and all the time, he pushed me.

A few weeks after I started my lectureship in sports medicine, he told me ‘Nick, bone always heals. Cartilage never heals. Where we can make a difference is in muscles, ligaments and tendons’. It was a no-brainer, and my interest in soft injuries was born. John made sure that I however undertook my ‘normal’ orthopaedic training, and in this way, I would never be shortsighted.

John was never short of advice. When I finished my Registrar training, and I was looking for a Senior Registrar post, he suggested that I applied outside of London. ‘People outside of London do not know you, but you are good. They will shortlist you, you will get great interview experience. But you will not be appointed, so by the time the right job will come in London you will blow them away with the answers you will have learnt from not getting the jobs outside of London’. I took his advice, and, not wanting to risk to not be in London, I applied to the Senior Registrar job as further away as possible, Aberdeen. Within 3 weeks of the application, I had been shortlisted. I spoke about it to Mr. King and to the other great John in my professional life, Mr. John Fixsen. They drilled me well, and 1 week later, I was in the far North East in the UK to be interviewed. I was offered the job. I did not know what to do and phoned Mr. King: ‘You bloody fool, just say yes’. And so another great part of my life came forward, and I moved to Scotland. At the time, in December 1992, there was no Internet, but I came down to London more often than not and kept in touch.

John told me that he would have supported my return to London, and he did try to establish a Senior Lectureship at the London with an interest in the upper limb. He came to visit me in Hong Kong, while I was an Associate Professor over there. On that occasion, Mrs. King, Deidre, told me to stop addressing her as ‘Mrs King’, and Mr. King asked me to address him as ‘John’. An honour I have been proud since.

Without the work that Prof. King pursued along the years, the UK would not have Sport and Exercise Medicine as a full-blown specialty: the course that he started in 1981 culminated a quarter of a century later with specialty recognition. To Prof. King the thanks from the whole Sports and Exercise Medicine community of the UK, whom he led when he was at the helm of the British Association of Sports Medicine.

Prof. King left his position of Senior Lecturer in Orthopaedic and Trauma Surgery in 2001 but has been present in the Centre for Sport and Exercise Medicine ever since; we made sure of that, and we continued to involve him by asking to lecture and examine on the course, to supervise research projects and to exert his clinical acumen with the patients the management of whom we did not have not a clue about!

In 2008, Mr. King convinced the powers that be that the Centre for Sport and Exercise Medicine needed a Professor. I successfully applied. When I took up post, in one congress, Mr. King commented publicly that I was now senior to him. This could not be, and in 2011, I made sure that Mr. King was granted an Honorary Professorship for his involvement with the course: a long due position for the man who led the British Association of Sport and Exercise Medicine and was awarded the Sir Roger Bannister Medal for Lifetime Services to Sports and Exercise Medicine.

Over the years, I developed more and more respect for the surgeon who trained me not just as a surgeon, but as a person and a doctor. I often cite Prof. King’s maxims and aphorisms and remember his smile and his wit. I sort of envy his style and teach my trainees what remains at the basis of what Prof. King instilled in me ‘Learn principles and concepts, not techniques. Principles stay, and you are great. Techniques change, and, without concepts, you are stuffed’.

Prof. King always maintained his family close: the love for his wife Deidre, for his children Polly and Toby and his grandchildren has always been foremost. And he has enlarged his family by embracing all the guys he trained, and at times their children. I know it: my own son, Giuseppe, is John’s godchild!

